# Mild-to-moderate severity of psoriasis may be assessed remotely based on photographs and self-reported extent of skin involvement

**DOI:** 10.1016/j.jdin.2023.02.004

**Published:** 2023-02-22

**Authors:** Zarqa Ali, John Robert Zibert, Priyanka Dahiya, Cæcilie Bachdal Johansen, Jesper Grønlund Holm, Astrid-Helene Ravn Jørgensen, Ionela Manole, Alina Suru, Alexander Egeberg, Simon Francis Thomsen, Anders Daniel Andersen

**Affiliations:** a Department of Dermato-Venereology and Wound Healing Centre, Copenhagen University Hospital Bispebjerg, Denmark; b Studies&Me A/S, Copenhagen, Denmark; c Future-Brain Aps, Copenhagen, Denmark; d Department of Dermatology, Dermatology Research Unit, Colentina Clinical Hospital, Bucharest, Romania; e Paediatric Dermatology Discipline, Dermatology Research Unit, Colentina Clinical Hospital; "Carol Davila" University of Medicine and Pharmacy, Bucharest, Romania; f Department of Biomedical Sciences, University of Copenhagen, Copenhagen, Denmark

**Keywords:** assessment, photographs, psoriasis, psoriasis vulgaris, severity, teledermatology

## Abstract

**Background:**

Remote monitoring was used to assess and manage skin diseases.

**Objective:**

To investigate to what extent smartphone photographs along with a self-reported body region (BR) score can be used to evaluate psoriasis severity.

**Methods:**

Psoriasis severity was assessed in the clinic using the psoriasis area and severity index and the physician’s global assessment. On the same day, the patients took a photograph of a representative lesion from 4 BR (head/neck, upper limbs, trunk, and lower limbs) and completed a questionnaire about BR score. The photographs were rated by 5 dermatologists. Intraclass correlation coefficients with 95% CIs were calculated.

**Results:**

Overall, 32 were included, of which 6% had almost clear, 69% had mild, and 25% had moderate psoriasis. Perfect agreement between the self-reported and the doctors’ BR score was observed for 59%, and near-perfect agreement (deviation of maximum 1 score) was 92%. The intraclass correlation coefficient between clinical and photographic psoriasis area and severity index was 0.78 (95% CI, 0.55-0.90), and for physician’s global assessment, perfect agreement was 53%.

**Conclusions:**

The agreement between psoriasis severity assessed clinically and by photographs was good in a study setting. This gives the opportunity to remotely assess psoriasis severity by combining photographs with self-reported BR scores.

**Capsule Summary**

• Remote monitoring is increasingly used to assess and manage psoriasis in remote clinical trials and e-consultations in the clinic.
• Combining photographs of psoriasis taken by a patient with self-reported body region score can be used to remotely assess psoriasis severity.

## Introduction

Psoriasis is a chronic inflammatory skin disease characterized by erythematous, scaly plaques often located on the extensor surfaces, elbows, knees, legs, scalp, and lumbosacral region.^1^^,^^2^ The worldwide prevalence of psoriasis is approximately 2%; however, the prevalence is lower in Asia and Africa, and higher in White and Scandinavian populations.^3^^,^^4^ Psoriasis can occur at any age, although a bimodal distribution pattern has been observed with an early onset approximately 20 to 30 years and a late onset approximately 50 to 60 years.^5^ Psoriasis is a clinical diagnosis; however, it has some characteristic histologic findings, including hyperkeratosis, parakeratosis, and acanthosis of the epidermis with dilated blood vessels and a lymphocytic infiltrate.^1^ Itching is the most characteristic symptom of psoriasis.^6^ A significant social and emotional burden is reported along with negative impact on physical well-being of patients with psoriasis affecting the quality of life substantially.^7^^,^^8^

Teledermatology, remote monitoring, e-health, and digital dermatology are expanding and increasingly used to assess and manage many skin diseases, including psoriasis.^9^ The assessment can either be performed based on patient-reported outcomes, photographs of skin lesions, or a combination of photographs and patient-reported outcomes. Furthermore, teledermatology has been shown to be as effective as in-person management in improving clinical outcomes among patients with psoriasis.^10^ Remote assessment is not only beneficial to replace, or as a supplement to a consultation with a doctor but may also be important for clinical trials enabling accurate and consecutive monitoring of disease severity and therapy response remotely.

In the present study, we investigated to what extent photographs of skin lesions taken by the patients along with a self-reported body region (BR) score performed when compared with established severity measures, ie, the psoriasis area and severity index (PASI) and physician’s global assessment (PGA) performed clinical (physical).

## Methods

### Recruitment

Individuals with psoriasis were recruited online using targeted Facebook advertisements. By clicking on the advertisement, individuals were guided to a study-specific landing page with participant information and opportunity to complete a screening questionnaire. All individuals passing the screening were sent a link to a booking system to book a time slot for an in-clinic visit. The diagnosis of psoriasis was confirmed on the day of examination in the clinic.

Individuals were included if they fulfilled all the inclusion criteria (age ≥18 years, diagnosis of psoriasis vulgaris confirmed by a physician at the in-clinic visit, active lesions of psoriasis vulgaris, and a smartphone with a functional camera) and none of the exclusion criteria (any other skin diseases influencing the evaluation of the psoriasis vulgaris severity, no visible psoriasis lesions, or patients only with scalp psoriasis at time of the study).

### Examination

Two independent assessments were performed in the clinic by 2 different physicians with different experience levels varying from board-certified dermatologists to dermatology resident doctors on the day of examination. The physicians completed PASI and PGA. Patients were onboarded to an Imagine photo-capturing application (LEO Innovation Lab) by the study staff.

On the day of examination, the patients were asked to take one photo of a representative lesion from each of the 4 anatomic regions (head/neck, upper limbs, trunk, and lower limbs) with their own smartphone from home. Later the same day patients received a link to an online questionnaire, via mobile text messages and email, about sleep, itch, and extent of psoriasis.

### Photographic assessment

All photographs taken by the patients were rated independently by the 5 board-certified dermatologists using secure browser-based and purpose-build dashboards on a tablet for the assessment. The photographic assessment was based on photographs taken by the patients with their own smartphone and self-reported BR score.

### Definitions

PASI comprises 3 components: erythema, induration, and scaling of psoriatic lesions each assessed on a 5-point scale: none (0), mild (1), moderate (2), severe (3), and very severe (4). Furthermore, it entails 4 measurements of the extent of skin involvement, splitting the body into 4 different anatomic regions (head and neck, upper limbs, trunk, and lower limbs), each of which are given an extent score of 0 to 6 depending on the percentage of affected skin (BR score: 1: 0%-9%; 2: 10%-29%; 3: 30%-49%; 4: 50%-69%; 5: 70%-89%, and 6: 90%-100%). The scores are then computed to a final PASI score, from 0 (no lesions) to 72 (maximum severity of lesions).^11^^,^^12^

The PGA assesses 3 characteristics globally (erythema, induration, and scaling), which are summed, divided by 3, and then rounded to the nearest integer, to calculate the final score. This ranges from clear (0), almost clear (1), mild (2), moderate (3), and severe (4).^13^^,^^14^ For statistical analysis, the scale was assigned scores of 0 to 4, for “clear” to “severe,” respectively.

### Statistics

Pearson’s correlations and intraclass correlation coefficients (ICCs) with 95% CIs were calculated to evaluate the degree of agreement between clinical and photographic assessments. The ICC estimates were based on a 2-way random-effects model, absolute agreement, and average measure.^15^ An ICC of >0.90, 0.75 to 0.90, 0.50 to 0.75, and <0.50 generally agreed to indicate excellent, good, moderate, and poor agreement, respectively.^15^ For photographic PASI, the severity scores were calculated based on the dermatologist-rated intensity based on the photographs taken by the patients combined with the patient-reported extent of skin involvement (BR score). The correlation was also calculated for only the intensity part of the PASI (erythema, induration, and scaling) without extent.

For photographic PGA, a PGA score was assigned to each photograph, and the maximum PGA per patient was then carried forward and averaged across the dermatologists.

### Ethics

The Regional Scientific Ethics Committee, Copenhagen, Denmark, was contacted about the study but did not find approval necessary because the study was purely observational. Patients who successfully completed all study tasks received a gift card for skincare products from Nøie.dk.

## Results

The Facebook advertisements ran 12 days from September 5 to 17, 2019 and 10 days from January 20 to 29, 2020. During the 22 days of active recruitment, 92 individuals showed interest in the study of which 53% (*n* = 49) booked a time slot for a visit in the clinic, and 42 (86%) showed up. Six were excluded because they did not upload any photographs from home. Thirteen percent of the patients who uploaded a photograph from home had Fitzpatrick skin type 2, 74% had Fitzpatrick skin type 3, 8% had Fitzpatrick skin type 4, and in 5% it was not possible to score Fitzpatrick skin type. After the visit in the clinic, 4 were excluded (3 did not have psoriasis and 1 did not complete the questionnaire), leaving 32 patients for inclusion in the study. Overall, 25 (78%) were women and 7 (22%) were men. The mean age of the included patients was 47 years (SD ±15), and one-third were between 51 and 60 years (*n* = 11). Based on the clinical PASI score, 6% (*n* = 2) had almost clear, 69% (*n* = 22) had mild, and 25% (*n* = 8) had moderate psoriasis. No patients were in the severe or very severe category (Table I).

**Table I:** Baseline characteristics of the study cohort of 32 patients with psoriasis

Characteristics	Psoriasis (*N* = 32)
Age	
Mean (SD)	47 (15)
19-30 y	5 (16)
31-40 y	3 (9)
41-50 y	8 (25)
51-60 y	11 (34)
61-70 y	4 (13)
71-80 y	1 (3)
Sex	
Women (%)	25 (78)
Men (%)	7 (22)
Age of onset	
Mean (SD)	23 (13)
Itch∗ NRS	
Median (range)	4 (1-10)
Sleep∗ NRS	
Median (range)	2 (0-8)
Dryness 0-4	
Median (range)	2 (0-3)
Self-reported extent of skin involvement†	
Head and neck, *n* (%)	
Score 0	12 (37)
Score 1	8 (25)
Score 2	4 (13)
Score 3	3 (9)
Score 4	4 (13)
Score 5	1 (3)
Score 6	0 (0)
Total score, mean (SD)	1.53 (1.54)
Upper limbs, *n* (%)	
Score 0	8 (25)
Score 1	11 (34)
Score 2	11 (34)
Score 3	2 (6)
Score 4	0 (0)
Score 5	0 (0)
Score 6	0 (0)
Total score, mean (SD)	1.30 (0.88)
Trunk, *n* (%)	
Score 0	21 (66)
Score 1	9 (28)
Score 2	1 (3)
Score 3	0 (0)
Score 4	0 (0)
Score 5	1 (3)
Score 6	0 (0)
Total score, mean (SD)	0.53 (1.00)
Lower limbs, *n* (%)	
Score 0	8 (25)
Score 1	7 (22)
Score 2	14 (44)
Score 3	2 (6)
Score 4	1 (3)
Score 5	0 (0)
Score 6	0 (0)
Total score, mean (SD)	1.50 (1.01)
Self-reported body surface area	
Mean (SD)	7 (6)
PASI, *n* (%)	
Almost clear	2 (6)
Mild	22 (69)
Moderate	8 (25)
Severe	0 (0)
Very severe	0 (0)

*NRS*, Numerical rating scale (0-10); *PASI*, psoriasis area and severity index.

∗ The last 3 days.

† In each region, the area is expressed as nil (0), 1%-9% (score 1), 10%-29% (score 2), 30%-49% (score 3), 50%-69% (score 4), 70%-89% (score 5), or 90%-100% (score 6).

### BR score

For the BR score, perfect agreement between the patients and the doctors was observed for 59%; in 25% of scores, the patients scored higher than the doctors and in 16% the patients scored lower than the doctors. Perfect or near-perfect agreement with the deviation of maximum one score was 92%.

Perfect agreement between the first and second doctors assessing the patients in the clinic was observed for 71%. Near-perfect agreement was 93%.

### Validity of PASI based on smartphone photographs

**Fig 1:**
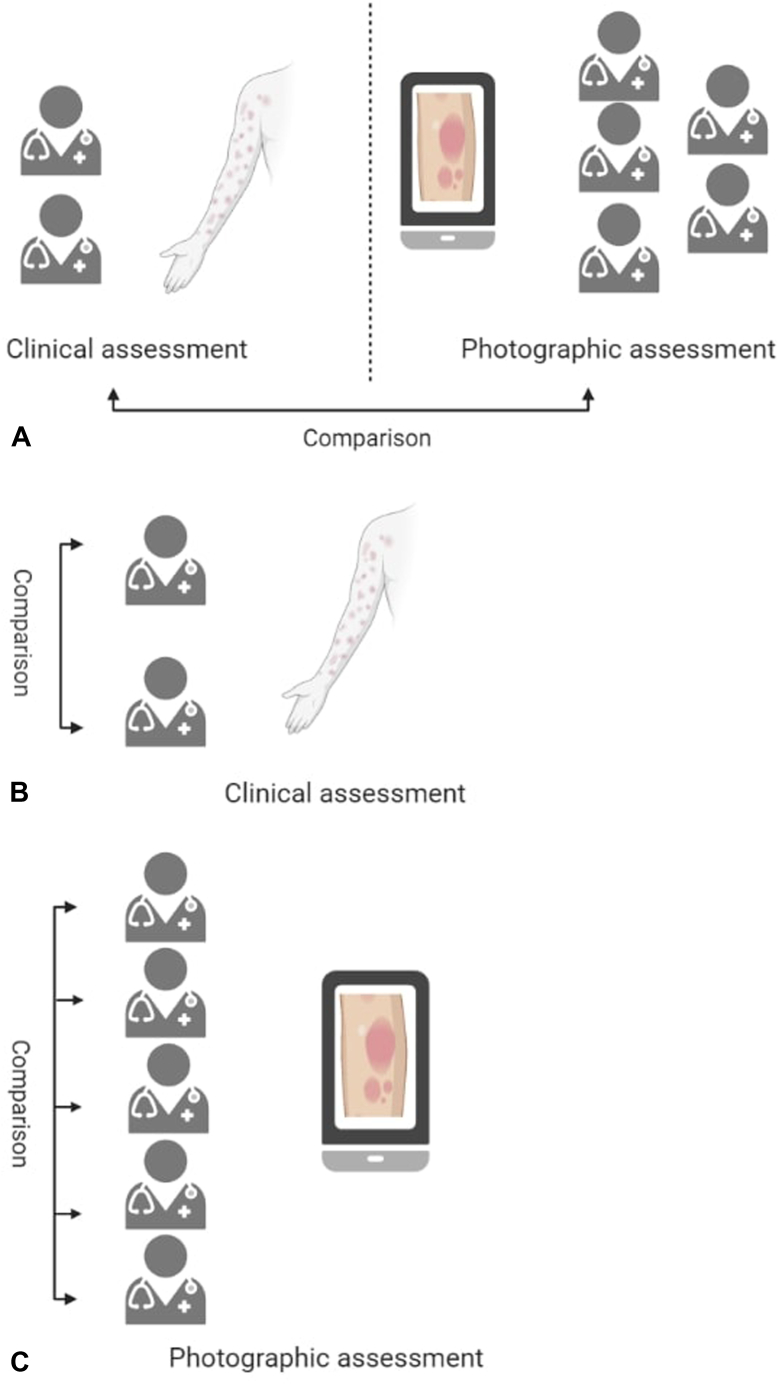
Illustration of the study design. **A**, comparison of clinical and photographic assessment. **B**, comparison between the 2 clinical assessors. **C**, comparison between the 5 photographic assessors.

The correlation between clinical assessment and photographic assessment (Fig 1, *A*) was *r* = 0.80. The ICC between clinical PASI and photographic PASI was 0.78 (95% CI, 0.55-0.90), and for the intensity part of the PASI, the ICC was 0.80 (95% CI, 0.60-0.90). The ICC was the highest for scaling (ICC, 0.72; 95% CI, 0.59-0.80) and lowest for erythema (ICC, 0.64; 95% CI 0.49-0.75) (Table II). For PGA, perfect agreement between clinical and photographic assessment was observed for 53%.

**Table II:** Comparison of erythema, induration, and scaling assessments based on photographs and clinical assessments

Characteristics	Comparison between clinical and photographic assessment ICC (95% CI)	Comparison between the first and second clinical assessment ICC (95% CI)	Comparison between different photographic assessments ICC (95% CI)
Erythema	0.641 (0.488-0.749)	0.880 (0.829-0.916)	0.983 (0.977-0.988)
Induration	0.649 (0.500-0.753)	0.825 (0.750-0.877)	0.970 (0.960-0.979)
Scaling	0.716 (0.594-0.801)	0.874 (0.821-0.912)	0.977 (0.968-0.984)

*ICC*, Intraclass correlation coefficient.

### PASI was assessed by 2 doctors in the clinic

The correlation between assessment from the first and second doctors in the clinic (Fig 1, *B*) was *r* = 0.92. The ICC between PASI assessed by the 2 doctors in the clinic was 0.92 (95% CI, 0.84-0.96), and for the intensity part of the PASI, the ICC was 0.87 (95% CI, 0.74-0.94). The ICC was the highest for erythema (ICC, 0.88; 95% CI, 0.83-0.92) (Table II). For PGA, perfect agreement between clinical and photographic evaluation was observed for 58%.

### Reliability of photographic assessment

The ICC for interrater reliability between the 5 dermatologists (Fig 1, *C*) was 0.99 (95% CI, 0.98-0.99) for photographic PASI and 0.91 (95% CI, 0.78-0.96) for the photographic intensity part of the PASI. The ICC was the highest for erythema (ICC, 0.98; 95% CI, 0.98-0.99) and lowest for induration (ICC, 0.97; 95% CI, 0.96-0.98) (Table II).

## Discussion

In this study, investigating the validity of photographic assessment of psoriasis, we recruited 32 patients within 22 days via targeted campaigns on Facebook. Self-reported rating of the BR score was comparable with the ratings given by the doctors. The agreement between the severity assessment of psoriasis made in the clinic and based on the photographs taken by the patients was overall good (ICC, 0.78), with the highest agreement observed for scaling and lowest for erythema. Further, severity assessment of psoriasis based on photographs was found to be reliable because the agreement was excellent between the 5 dermatologists’ severity ratings using PASI based on the photographs, ICC 0.99. Combining photographs with self-reported BR score can be used to remotely assess psoriasis severity.

In remote severity assessment of psoriasis based on PASI, the doctor will rely on the patient-reported BR score because it is very difficult to capture the entire body surface area on photographs from home for the doctor to make an assessment. Between doctors, a significant interrater reliability issue regarding the measure of affected skin has been described.^12^ In our study, perfect agreement regarding BR score between the patients and the doctors was observed for 59%, and for 71% between the first and second doctor assessing the psoriasis severity in the clinic. Perfect or near-perfect agreement with a deviation of maximum one score was 92% between the patients and doctor, and 93% between the 2 clinical doctors. These agreements are very close, although the patient tended to score higher than the doctor in 1 out of 4 cases and lower in 1 out of 5 cases. With proper patient education, it might be possible to reach an even higher agreement level between the BR score evaluated by the patient and the doctor, making the severity assessment based on remote PASI more accurate. The agreement between total PASI was very close to the agreement between the intensity part of the PASI because the BR score was so well correlated with the doctor’s assessments. It emphasizes the importance of patient education when responsibilities are shifted from the doctor to the patient. In teledermatologic solutions and remote ratings of skin diseases, a much of the responsibility will reside with the patient. In clinical assessment the evaluation is based on the doctor’s observation; however, for remote assessments the doctor relies on the data provided by the patients. The more accurate data the patients provide, the closer the assessment comes to the clinical assessment. In a study with patients with atopic dermatitis, we have shown that the interrater ICCs for photographic eczema area and severity index assessment and photographic SCOring Atopic Dermatitis assessment compared with clinical assessment, were 0.90 and 0.96, respectively.^16^ In this study, the photographic assessment was based on photographs taken by the patients and the extent evaluated in the clinic by the doctors. The agreement is a bit lower for psoriasis than for eczema, likely because both the photographs and the extent were provided by the patients.

Both PASI and PGA are simple measurement tools to rate the severity of psoriasis commonly used in clinical practice and particularly in clinical trials.^1^ It is well known that PASI scores can vary substantially between experienced and inexperienced physicians, raising concerns for interrater reliability, even in traditional in-clinic assessment.^14^ Although, the ICC for interrater and intrarater reliability for PASI has been described to be between good to excellent (ICC between 0.7 and >0.9) in the literature.^17^, ^18^, ^19^, ^20^ In the present study, the ICC was rated to be excellent between the 2 clinical assessments 0.92. The ICC was a bit lower though still good when photographic PASI assessment was compared with clinical PASI assessment 0.78. However, looking at the components of the intensity part of the PASI (erythema, induration, and scaling) the ICC was highest for the interrater reliability between the 5 dermatologists rating the photographs. This was even higher than the ICC between photographic and clinical assessment and ICC between the 2 clinical assessors. One explanation could be that the interrater reliability based only on photographs was made by 5 dermatologists, in contrast to the clinical assessment made by doctors with different clinical experience levels varying from dermatologists to resident doctors. The high level of experience could thereby be an explaining factor for the higher interrater reliability based on photographs. Another explanation could be that when assessing the photographs, all raters were assessing the same lesion photographed by the patients. In another study with atopic dermatitis, we found a good agreement among dermatologists in the selection of the most representative area and characteristics for severity assessment on a photograph, regardless of their experience level in dermatology and with photo-based severity assessment.^21^ In a clinical setting, the evaluating doctor is choosing a target lesion to be assessed, and there may be a difference in the choice of a target lesion resulting in a lower agreement. However, the agreement for erythema, induration, and scaling was higher between the 2 clinical assessors than the photographic assessment compared with clinical assessment. Although the 2 clinical assessors themselves had chosen the target lesion. The slightly lower agreement between photographic assessment and clinical assessment could also be explained by the fact that the photographs were chosen by the patients. Patients may tend to choose a severe lesion as a target lesion and thereby not the average lesion as a target lesion. Better patient education in choosing a target lesion will help overcome this challenge.

Finally, the agreement for interrater reliability between the 5 dermatologists for an overall PASI was excellent (0.99). However, for all these 5 ratings, the patient-reported extent was the same. Because this constant was the same throughout the 5 ratings, it will lead to a better agreement. Although, when ignoring this constant and only considering the intensity part of the PASI without BR score, the agreement was still higher between the 5 dermatologists compared with the agreement between clinical and photographic assessment (0.91 vs 0.80).

Further, the study had important strengths and limitations that need to be addressed. With respect to the strengths, first, the study was designed to validate photographic assessment of psoriasis, including both photographs and self-reported extent of skin involvement, to examine the remote assessment entirely relying on the patient. Second, both PASI, PGA, and BR scores were assessed in the study. Third, patients were recruited online and not already known by the clinical assessors preventing bias. Fourth, both board-certified dermatologists and resident doctors evaluated the patients. In contrast, the limitations were that severe and very severe cases were not included in the study. PASI in general is criticized for having a narrow band of scores, thereby decreasing the usefulness of the full range of scores (because scores >40 are rare), further it is not good to discriminate at lower scores.^12^ The validity of the scale is therefore believed to be overrated, in part because of the skewing toward lower scores.^22^ Second, there was a difference in the level of experience between the doctors performing the in-clinic assessment. Finally, the fact that the patients were aware of they were participating in a study exploring photographic assessment of skin lesions could have an impact on their consciousness on the quality of the photographs, which gives the physicians optimal condition to assess the skin disease. This may not be the case in real-life when the physicians receive photographs from the patients in situations where in-person examination is not possible. However, this issue can be overcome by educating patients in how to capture a good photograph of a skin lesion regarding lighting, resolution, and focus.

In conclusion, there is a good agreement between the severity of psoriasis assessed on photographs taken by the patient with a smartphone of a target lesion in combination with self-reported extent of skin involvement and the clinical assessment of psoriasis. The use of photographs in the evaluation of psoriasis severity is also reliable because the agreement between dermatologists was excellent. Future studies should investigate the validation of photographic assessment in the entire range of psoriasis severity and across all skin types and ethnicities.

## Conflicts of interest

Drs JRZ, PD, ADA, IM, and AS were employed by Studies&Me at the time of study execution. Drs ZA, JGH, ARJ, AE, SFT, and CBJ have no conflicts of interest to declare regarding this paper.

## References

[1] Kimmel G.W., Lebwohl M. (2018). Psoriasis: overview and diagnosis. Evidence-Based Psoriasis.

[2] Egeberg A., Griffiths C.E.M., Williams H.C., Andersen Y.M.F., Thyssen J.P. (2020). Clinical characteristics, symptoms and burden of psoriasis and atopic dermatitis in adults. Br J Dermatol.

[3] Parisi R., Symmons D.P., Griffiths C.E., Ashcroft D.M. (2013). Identification and Management of Psoriasis and Associated ComorbidiTy (IMPACT) project team. Global epidemiology of psoriasis: a systematic review of the incidence and prevalence. J Invest Dermatol.

[4] Egeberg A., Andersen Y.M.F., Thyssen J.P. (2019). Prevalence and characteristics of psoriasis in Denmark: findings from the Danish skin cohort. BMJ Open.

[5] Henseler T., Christophers E. (1985). Psoriasis of early and late onset: characterisation of two types of psoriasis vulgaris. J Am Acad Dermatol.

[6] Lebwohl M.G., Bachelez H., Barker J. (2014). Patient perspectives in the management of psoriasis: results from the population-based multinational assessment of Psoriasis and Psoriatic Arthritis Survey. J Am Acad Dermatol.

[7] Pariser D., Schenkel B., Carter C. (2016). A multicenter, non-interventional study to evaluate patient-reported experiences of living with psoriasis. J Dermatolog Treat.

[8] Krueger G., Koo J., Lebwohl M., Menter A., Stern R.S., Rolstad T. (2001). The impact of psoriasis on quality of life: results of a 1998 National Psoriasis Foundation patient-membership survey. Arch Dermatol.

[9] Havelin A., Hampton P. (2022). Telemedicine and e-health in the management of psoriasis: improving patient outcomes - a narrative review. Psoriasis (Auckl).

[10] Armstrong A.W., Chambers C.J., Maverakis E. (2018). Effectiveness of online vs in-person care for adults with psoriasis: a randomized clinical trial. JAMA Netw Open.

[11] Mattei P.L., Corey K.C., Kimball A.B. (2014). Psoriasis area severity index (PASI) and the dermatology life quality index (DLQI): the correlation between disease severity and psychological burden in patients treated with biological therapies. J Eur Acad Dermatol Venereol.

[12] Ashcroft D.M., Wan Po A.L., Williams H.C., Griffiths C.E. (1999). Clinical measures of disease severity and outcome in psoriasis: a critical appraisal of their quality. Br J Dermatol.

[13] Cappelleri J.C., Bushmakin A.G., Harness J., Mamolo C. (2013). Psychometric validation of the physician global assessment scale for assessing the severity of psoriasis disease activity. Qual Life Res.

[14] Langley R.G., Ellis C.N. (2004). Evaluating psoriasis with psoriasis area and severity index, psoriasis global assessment, and lattice system physician's global assessment. J Am Acad Dermatol.

[15] Koo T.K., Li M.Y. (2016). A guideline of selecting and reporting intraclass correlation coefficients for reliability research. J Chiropr Med.

[16] Ali Z., Chiriac A., Bjerre-Christensen T. (2022). Mild-to-moderate atopic dermatitis severity can be reliably assessed using smartphone-photographs taken by the patient at home: a validation study. Skin Res Technol.

[17] Berth-Jones J., Thompson J., Papp K. (2008). A study examining inter-rater and intrarater reliability of a novel instrument for the assessment of psoriasis: the Copenhagen Psoriasis Severity Index. Br J Dermatol.

[18] Cabrera S., Chinniah N., Lock N., Cains G.D., Woods J. (2015). Inter-observer reliability of the PASI in a clinical setting. Australas J Dermatol.

[19] Berth-Jones J., Grotzinger K., Rainville C. (2006). A study examining inter- and intrarater reliability of three scales for measuring the severity of psoriasis: psoriasis area and severity index, physician's global assessment, and lattice system physician's global assessment. Br J Dermatol.

[20] Bożek A., Reich A. (2017). The reliability of three psoriasis assessment tools: psoriasis area and severity index, body surface area, and physician global assessment. Adv Clin Exp Med.

[21] Ali Z., Andersen A.D., Bjerre-Christensen T., Libert J.R., Thomsen S.F. (2021). Agreement between dermatologists' selection of the lesional area and characteristics best representing atopic dermatitis severity: online survey. J Am Acad Dermatol.

[22] Gouraud P.A., Le Gall C., Puzenat E., Aubin F., Ortonne J.P., Paul C.F. (2012). Why statistics matter: limited inter-rater agreement prevents using the psoriasis area and severity index as a unique determinant of therapeutic decisions in psoriasis. J Invest Dermatol.

